# Supplementary material for: Wild-Type KRAS Allele Effects on Druggable Targets in KRAS Mutant Lung Adenocarcinomas
Source: Genes (Basel). 2021 Sep 11;12(9):1402. doi: 10.3390/genes12091402 (PMC8467269; doi:10.3390/genes12091402)

Table S1: List of antibodies used for RPPA analysis including target proteins, vendor, species, dilution, and p values for two-group comparisons between *KRASm/WT+* and *KRASm/WT-* NSCLC cell lines.

Antibody	Vendor	Catalog number	Dilution	Host	p-value
4E-BP1 S65	CellSig	9451	1:50	R	0.14
4E-BP1 T70	CellSig	9455	1:200	R	0.32
Acetyl-CoA Carboxylase S79	CellSig	3661	1:50	R	**< 0.01**
AKT S473	CellSig	9271	1:100	R	0.16
Alk	CellSig	3633	1:50	R	**< 0.01**
Alk 1604	CellSig	3341	1:50	R	0.20
AMPK alpha T172	CellSig	4188	1:2000	R	0.20
AMPK alpha1 S485	CellSig	4184	1:50	R	0.75
AMPK beta1 S108	CellSig	4181	1:50	R	0.17
Androgen Receptor S81	Millipore	07-1375	1:1000	R	0.41
A-Raf S299	CellSig	4431	1:50	R	0.10
ATF-2 T69/71	CellSig	9225	1:500	R	0.19
ATG5	CellSig	2630	1:1000	R	0.59
ATG12	CellSig	2010	1:100	R	0.52
ATM S1981	CellSig	5883	1:50	R	**< 0.01**
ATP-Citrate Lyase S454	CellSig	4331	1:100	R	0.20
ATR S428	CellSig	2853	1:50	R	0.08
Aurora A T288/Aurora B T232/Aurora C T198	CellSig	2914	1:50	R	0.39
Axl Y702	CellSig	5724	1:50	R	**< 0.01**
BAD S112	CellSig	9291	1:200	R	**< 0.01**
BAD S136	CellSig	9295	1:50	R	0.05
BAD S155	CellSig	9297	1:100	R	0.92
Bak	CellSig	3814	1:100	R	**< 0.01**
Bax	CellSig	2772	1:200	R	0.05
Bcl-2 S70	CellSig	2827	1:50	R	0.97
Bcl-2 T56	CellSig	2875	1:200	R	0.72
Bcl-xL	CellSig	2762	1:500	R	0.95
Beclin1	CellSig	3738	1:100	R	**0.03**
BIM	CellSig	2933	1:500	R	0.13
B-Raf S445	CellSig	2696	1:50	R	**< 0.01**
c-Met	Abcam	ab51067	1:200	R	0.96
c-Abl T735	CellSig	2864	1:50	R	1.00
c-Abl Y245	CellSig	2861	1:100	R	**< 0.01**
Caspase-3, cleaved (D175)	CellSig	9661	1:50	R	0.96

Caspase-6, cleaved (D162)	CellSig	9761	1:50	R	0.15
Caspase-7, cleaved (D198)	CellSig	9491	1:100	R	0.40
Caspase-9, cleaved (D330)	CellSig	9501	1:50	R	**< 0.01**
Catenin beta S33/37/T41	CellSig	9561	1:100	R	0.12
Chk1 S345	CellSig	2341	1:50	R	**0.04**
Chk2 S33/35	CellSig	2665	1:50	R	0.40
Cofilin S3	CellSig	3313	1:500	R	**< 0.01**
Cox-2	BD	610203	1:200	M	**< 0.01**
cPLA2 S505	CellSig	2831	1:1000	R	**0.02**
C-Raf S338	CellSig	9427	1:200	R	**< 0.01**
CREB S133	CellSig	9191	1:100	R	0.98
CrkL Y207	CellSig	3181	1:100	R	**0.03**
Cyclin A2	CellSig	4656	1:50	M	**0.03**
Cyclin B1	CellSig	4135	1:200	M	0.11
Cyclin D1	BD	554180	1:100	M	**0.04**
EGFR	CellSig	2232	1:100	R	0.52
EGFR Y1045	CellSig	2237	1:50	R	0.29
EGFR Y1068	CellSig	2234	1:50	R	0.88
EGFR Y1148	BioSource	44-792	1:100	R	**0.02**
EGFR Y1173	BioSource	44-794	1:100	R	0.16
eIF4E S209	CellSig	9741	1:50	R	0.22
eIF4G S1108	CellSig	2441	1:1000	R	0.27
Elk-1 S383	CellSig	9181	1:100	R	**0.03**
eNOS S113	CellSig	9575	1:50	R	0.46
eNOS/NOS III S116	Upstate	07-357	1:500	R	**< 0.01**
Ephrin A3 Y799/A4 Y799/A5 Y833	Abcam	124881	1:100	R	0.93
ERK 1/2 T202/Y204	CellSig	9101	1:1000	R	**0.04**
Estrogen Receptor alpha	CellSig	2511	1:1000	M	0.26
Estrogen Receptor alpha S118	CellSig	2511	1:1000	M	0.11
Etk Y40	CellSig	3211	1:2000	R	0.17
Ezrin T567/Radixin T564/Moesin T558	CellSig	3141	1:100	R	**< 0.01**
FADD S194	CellSig	2781	1:100	R	**< 0.01**
FAK Y576/577	CellSig	3281	1:200	R	**< 0.01**
FOXO1 T24/FOXO3 T32	CellSig	9464	1:200	R	**< 0.01**
FOXO3 S253	Upstate	06-953	1:1000	R	0.06
FOXM1 T600	CellSig	14655	1:100	R	**< 0.01**
FOXO1 S256	CellSig	9461	1:100	R	**< 0.01**
Grb2	CellSig	3972	1:1000	R	0.36
GSK-3 alpha/beta S21/9	CellSig	9331	1:100	R	0.96
HDAC 1	CellSig	2062	1:100	R	**< 0.01**

HDAC 3	CellSig	2632	1:1000	R	**0.01**
HDAC 4	CellSig	2072	1:100	R	**< 0.01**
HDAC 6	SantaCruz	sc-11420	1:2000	R	**0.04**
HER2	DAKO	A0485	1:250	R	0.35
HER2 Y1248	Imgenex	IMG-90189	1:500	R	0.86
HER3 Y1197	CellSig	4561	1:100	R	**< 0.01**
HER3 Y1289	CellSig	4791	1:200	R	0.91
Histone H3, Acetyl Lys9 14	CellSig	9677	1:2000	R	0.22
Histone H3, Di-Methyl Lys9	CellSig	9753	1:500	R	0.30
Histone H3, Pan-Methyl Lys9	CellSig	4069	1:100	R	**< 0.01**
Histone H3 S10	Upstate	06-570	1:200	R	**0.02**
Histone H3 S28	Upstate	07-145	1:1000	R	0.28
Histone H4 Acetyl Lys8	CellSig	2594	1:200	R	**< 0.01**
HSP27 S82	CellSig	2406	1:100	R	0.22
HSP90a T5/7	CellSig	3488	1:100	R	0.34
IGF-1R Y1131 IR Y1146	CellSig	3021	1:500	R	0.07
IGF-1R Y1135/1136 IR Y1150/1151	CellSig	3024	1:500	R	0.85
IkappaB-alpha S32/36	CellSig	9246	1:100	M	**< 0.01**
IL-8	Abcam	ab7747	1:200	R	0.88
IL-10	Abcam	ab52909	1:2000	R	0.06
IL-11	SantaCruz	sc-7924	1:500	R	0.06
Insulin Receptor beta	CellSig	3025	1:200	R	0.23
IRS-1 S612	CellSig	2386	1:200	R	0.66
Jak1 Y1022/1023	CellSig	3331	1:50	R	**0.01**
Ki67	DAKO	M7240	1:100	M	**< 0.01**
LC3B	CellSig	2775	1:100	R	0.22
Lck Y505	Biosource	44-850	1:50	R	0.07
LIMK1 T508/LIMK2 T505	CellSig	3841	1:100	R	0.82
LKB1 S334	CellSig	3055	1:50	R	**0.02**
LKB1 S428	CellSig	3051	1:100	R	0.39
MARCKS S152/156	CellSig	2741	1:200	R	0.11
MDM2 S166	CellSig	3521	1:100	R	0.98
MEK 1/2 S217/221	CellSig	9121	1:200	R	**< 0.01**
Met Y1234/1235	CellSig	3126	1:200	R	**< 0.01**
MSK1 S360	CellSig	9594	1:50	R	0.65
mTOR S2448	CellSig	2971	1:100	R	**< 0.01**
NF-kappaB p65 S536	CellSig	3031	1:100	R	**< 0.01**
p27/Kip1	BD	610242	1:100	M	**< 0.01**
p27 T187	Zymed	71-7700	1:200	R	**0.02**
p38 MAP Kinase T180/Y182	CellSig	9211	1:100	R	0.88

p53	CellSig	9282	1:5000	R	0.13
p53 S15	CellSig	9284	1:1000	R	**< 0.01**
p62/SQSTM1	CellSig	8025	1:50	R	**< 0.01**
p70 S6 Kinase S371	CellSig	9208	1:50	R	0.63
p70 S6 Kinase T389	CellSig	9205	1:100	R	**< 0.01**
p70 S6 Kinase T412	Upstate	07-018	1:500	R	**< 0.01**
p90RSK S380	CellSig	9341	1:200	R	0.32
p90RSK T359/S363	CellSig	9344	1:200	R	0.88
PAK1 S199/204 PAK2 S192/197	CellSig	2605	1:50	R	**0.04**
PAK1 T423/PAK2 T402	CellSig	2601	1:100	R	0.29
PARP, cleaved (D214)	CellSig	9541	1:100	R	**< 0.01**
Paxillin Y118	CellSig	2541	1:500	R	**0.01**
PDGF Receptor beta Y751	CellSig	3161	1:50	R	0.24
PDK1 S241	CellSig	3061	1:200	R	**0.03**
PDL1 E1L3N	CellSig	13684	1:500	R	0.13
PI3 Kinase p110gamma	CellSig	4252	1:100	R	**0.01**
PIAS1	CellSig	3550	1:100	R	0.39
PKA C T197	CellSig	4781	1:200	R	0.17
PKC a/BII T638/641	CellSig	9375	1:100	R	0.39
PKC alpha S657	Upstate	06-822	1:1000	R	0.98
PKC delta T505	CellSig	9374	1:50	R	0.08
PKC theta T538	CellSig	9377	1:100	R	**< 0.01**
PKC zeta/lambda T410/403	CellSig	9378	1:50	R	0.40
PLCgamma1 Y783	CellSig	2821	1:100	R	0.57
PLK1 T210	BD	558400	1:200	M	0.57
PP2A A Subunit	CellSig	2039	1:1000	R	**< 0.01**
PP2A B Subunit	CellSig	4953	1:1000	R	**0.03**
PRAS40 T246	BioSource	44-1100	1:1000	R	0.13
PTEN	CellSig	9552	1:50	R	0.39
PTEN S380	CellSig	9551	1:500	R	0.72
Pyk2 Y402	CellSig	3291	1:200	R	**< 0.01**
Raf S259	CellSig	9421	1:100	R	**< 0.01**
Ras-GRF1	CellSig	3322	1:200	R	**< 0.01**
Ras-GRF1 S916	CellSig	3321	1:50	R	0.09
Rb S780	CellSig	3590	1:2000	R	**0.02**
Ret Y905	CellSig	3221	1:100	R	0.92
Ron Y1353	Epitomics	5176-1	1:1000	R	0.20
RSK3 T356/S360	CellSig	9348	1:500	R	**< 0.01**
S6 Ribosomal Protein S235/236	CellSig	4856	1:200	R	0.25
S6 Ribosomal Protein S240/244	CellSig	2215	1:1000	R	0.22

SAPK/JNK T183/Y185	CellSig	9251	1:100	R	**0.03**
SEK1/MKK4 S80	CellSig	9155	1:50	R	**< 0.01**
SGK1 S78	CellSig	5599	1:100	R	0.46
Shc Y317	Upstate	07-206	1:200	R	**< 0.01**
SHIP1 Y1020	CellSig	3941	1:50	R	0.40
SHP2 Y580	Biosource	44-558	1:500	R	**< 0.01**
Smad2 S245/250/255	CellSig	3104	1:100	R	**< 0.01**
Smad2 S465/467	CellSig	3101	1:200	R	**< 0.01**
SOCS3	CellSig	2923	1:50	R	0.78
SOCS1	CellSig	3950	1:50	R	**< 0.01**
Src Family Y416	CellSig	2101	1:100	R	**< 0.01**
Src Y527	CellSig	2105	1:200	R	**< 0.01**
Stat1 Y701	CellSig	9171	1:500	R	0.79
Stat2 Y690	CellSig	4441	1:100	R	0.79
Stat3 Y705	CellSig	9145	1:100	R	**< 0.01**
Stat3 S727	CellSig	9134	1:100	R	0.07
Stat4 Y693	CellSig	5267	1:100	R	**< 0.01**
Stat5 Y694	CellSig	9351	1:50	R	0.09
Stat6 Y641	CellSig	9361	1:100	R	0.22
Survivin	CellSig	2808	1:500	R	0.20
Syk Y525/526	CellSig	2711	1:50	R	**< 0.01**
TGF-Beta	CellSig	3709	1:1000	R	**0.03**
TNF alpha	Abcam	ab9635	1:200	R	**< 0.01**
TNF-R1	CellSig	3736	1:50	R	**0.03**
Tubulin alpha	Sigma	T 6074	1:2000	M	**< 0.01**
Tyk2	CellSig	9312	1:200	R	0.66
VASP S157	CellSig	3111	1:100	R	0.72
XIAP	CellSig	2042	1:100	R	**0.02**
YAP S127	CellSig	13008	1:100	R	**< 0.01**

Figure S1: Bar graphs illustrating phosphorylation levels of apoptotic proteins on residues regulating proteins cytosolic sequestration and inactivation in *KRASm/WT-* and *KRASm/WT+* adenocarcinoma cell lines.

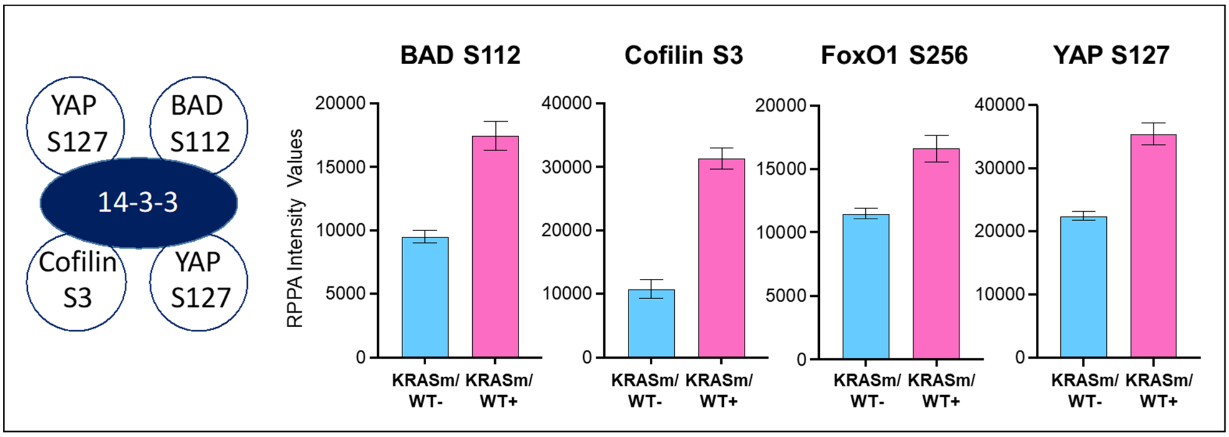

Supplement: Supplementary file 1 [file genes-12-01402-s001.zip › genes-1349275-supplementary.pdf]
